# P-209. The Burden of Crimean Congo Hemorrhagic Fever in the Military Health System

**DOI:** 10.1093/ofid/ofaf695.431

**Published:** 2026-01-11

**Authors:** Ryan B Liberg, Alison Helfrich, Saira Shaikh, Patrick Hickey, David A Lindholm

**Affiliations:** Brooke Army Medical Center, Fort Sam Houston, TX; Uniformed Services University of the Health Sciences, Bethesda, Maryland; Infectious Disease Clinical Research Program, Department of Preventive Medicine and Biostatistics, Uniformed Services University of the Health Sciences, Bethesda, MD, USA, Bethesda, Maryland; Uniformed Services University, Bethesda, Maryland; Uniformed Services University of the Health Sciences, Bethesda, Maryland

## Abstract

**Background:**

Crimean Congo Hemorrhagic Fever (CCHF) virus is an arborvirus with a clinical spectrum ranging from asymptomatic infection to severe hemorrhagic fever. Due to its endemicity in regions of US military operational interest, it poses a potential threat to military readiness, but its burden is poorly defined. We sought to estimate the burden of CCHF within the Military Health System (MHS).

**Figure:**
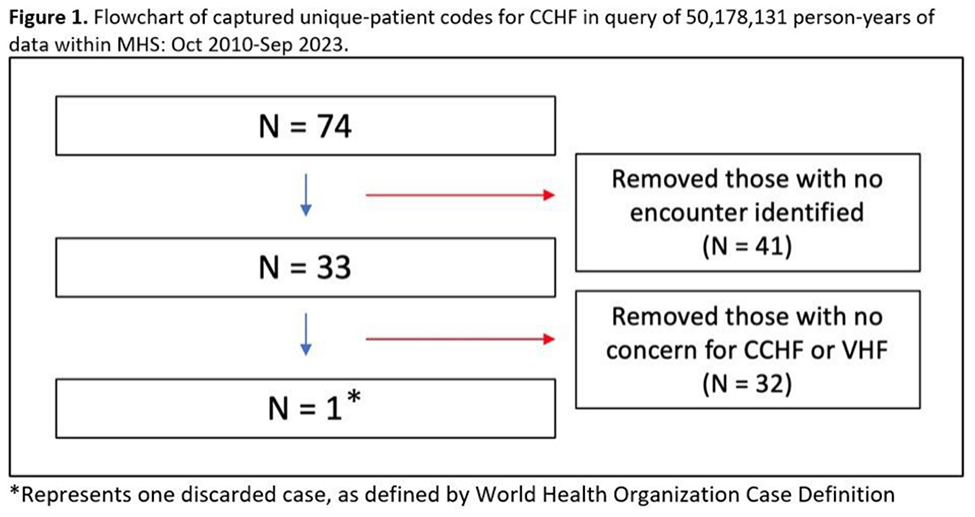

**Methods:**

The Deployment and Travel Health: Knowledge, Attitudes, Practices, and Outcomes Study (KAPOS) evaluates travel-associated diseases in the MHS. We searched the MHS Data Repository for International Classification of Diseases (ICD) 9/10 codes and Logical Observation Identifier Names and Codes (LOINC) for CCHF in military beneficiaries receiving care in military treatment facilities (direct care) or civilian centers (purchased care) from Oct 2010-Sep 2023. For diagnostic validation, charts were reviewed for encounters within 6 months of captured codes using the World Health Organization (WHO) case definition and clinical context.

**Results:**

We queried 50,178,131 person-years of data, identifying 74 unique-patient codes for CCHF: 73 by ICD code (all purchased care) and 1 by LOINC (direct care). Chart review excluded 41 codes due to no temporally associated clinical encounters available within the electronic medical record of the MHS, while 32 others had no concern for CCHF nor another viral hemorrhagic fever (VHF), reflecting incorrect ICD coding. The single code captured via LOINC was deemed a discarded case by the WHO case definition, despite clinical suspicion for CCHF.

**Conclusion:**

While suspected cases of CCHF in the MHS were rare, its potential impact remains uncertain. The ICD codes for CCHF in our system captured exclusively purchased care, limiting the availability of records for review. The lack of clinical concern for hemorrhagic fever in ICD-coded charts underscores the low sensitivity of ICD codes in estimating the burden of rare infections, whereas the use of LOINC may increase the probability of detecting a clinically relevant code. Alternative methods for characterizing burden should be considered (e.g., seroprevalence survey in those with risk factors) given its rarity, spectrum of clinical presentations, and operational exposure risk.

**Disclosures:**

All Authors: No reported disclosures

